# Supplementary material for: Protocol: Developing a framework to improve glycaemic control among patients with type 2 diabetes mellitus in Kinshasa, Democratic Republic of the Congo
Source: PLoS One. 2022 Sep 26;17(9):e0268177. doi: 10.1371/journal.pone.0268177 (PMC9512168; doi:10.1371/journal.pone.0268177)
Supplement: S2 Appendix — (DOCX) [file pone.0268177.s002.docx]

|  | |  | **STUDY TITLE:** DEVELOPING A FRAMEWORK TO IMPROVE GLYCAEMIC CONTROL AMONG PATIENTS WITH TYPE 2 DIABETES MELLITUS IN KINSHASA, DEMOCRATIC REPUBLIC OF THE CONGO  **S2 APPENDIX: QUESTIONNAIRE** | | | | | | | | | | | | | |  | |  |  |  |  |  |
| --- | --- | --- | --- | --- | --- | --- | --- | --- | --- | --- | --- | --- | --- | --- | --- | --- | --- | --- | --- | --- | --- | --- | --- |
|  | |  | **ID:……………………………… Province:……………………** | | | | | | | | | | | | | |  | |  |  |  |  |  |
|  | |  | **Place:………………………..... Investigator number:…………**  **Date: / /** | | | | | | | | | | | | | |  |  |  |  |  |  |  |
|  | |  | **Sociodemographic characteristics** | | | | | | | | | | | | | |  |  |  |  |  |  |  |
| **1** | |  | Age (years):…………….. | | |  | | | | |  | | | | | |  |  |  |  |  |  |  |
| **2** | |  | Sex: | | |  | | | | |  | | | | | |  |  |  |  |  |  |  |
|  | |  |  | | 1. Male | | | | | |  | | | | | |  |  |  |  |  |  |  |
|  | |  |  | | 2. Female | | | | | |  | | | | | |  |  |  |  |  |  |  |
| **3** | |  | Marital status: ....................... | |  | | | | | |  | | | | | |  |  |  |  |  |  |  |
|  | |  |  | | 1. Single | | | | | |  | | | | | |  |  |  |  |  |  |  |
|  | |  |  | | 2. Free union | | | | | |  | | | | | |  |  |  |  |  |  |  |
|  | |  |  | | 3. Married (monogamic) | | | | | |  | | | | | |  |  |  |  |  |  |  |
|  | |  |  | | 4. Married (polygamic) | | | | | |  | | | | | |  |  |  |  |  |  |  |
|  | |  |  | | 5. Divorced/ Separated | | | | | |  | | | | | |  |  |  |  |  |  |  |
|  | |  |  | | 6. Widow | | | | | |  | | | | | |  |  |  |  |  |  |  |
| **4** | |  | Ethnic group:…………………... | | 1.Kongo | | | | | |  | | | | | |  |  |  |  |  |  |  |
|  | |  |  | | 2. Luba | | | | | |  | | | | | |  |  |  |  |  |  |  |
|  | |  |  | | 3. Ngala | | | | | |  | | | | | |  |  |  |  |  |  |  |
|  | |  |  | | 4. Swahili | | | | | |  | | | | | |  |  |  |  |  |  |  |
|  | |  |  | | 5. Other………………………… | | | | | |  | | | | | |  |  |  |  |  |  |  |
| **5** | |  | Religion:……………………… | |  | | | | | |  | | | | | |  |  |  |  |  |  |  |
|  | |  |  | | 1. Catholic | | | | | |  | | | | | |  |  |  |  |  |  |  |
|  | |  |  | | 2. Protestant  3. Independent group | | | | | |  | | | | | |  |  |  |  |  |  |  |
|  | |  |  | | 4. Jehovah witness | | | | | |  | | | | | |  |  |  |  |  |  |  |
|  | |  |  | | 5. Kimbanguist | | | | | |  | | | | | |  |  |  |  |  |  |  |
|  | |  |  | | 5. Muslim | | | | | |  | | | | | |  |  |  |  |  |  |  |
|  | |  |  | | 6. Animist | | | | | |  | | | | | |  |  |  |  |  |  |  |
|  | |  |  | | 7. Without religion  8. Other………………… | | | | | |  | | | | | |  |  |  |  |  |  |  |
| **6** | |  | Highest general education qualification: | |  | | | | | | | | | | | |  |  |  |  |  |  |  |
|  | |  |  | | 1.No formal education  2. Primary school | | | | | | | |  | | | |  |  |  |  |  |  |  |
|  | |  |  | | 3. Secondary school | | | | | | | |  | | | |  |  |  |  |  |  |  |
|  | |  |  | | 4. University | | | | | | | |  | | | |  |  |  |  |  |  |  |
| **7** | |  | Occupation | |  |  | | | | | | | | | | |  |  |  |  |  |  |  |
|  | |  |  | | 1. Employed | | | | |  | | | | | | |  |  |  |  |  |  |  |
|  | |  |  | | 2. Unemployed | | | | |  | | | | | | |  |  |  |  |  |  |  |
|  | |  |  | | 3. Other | | | | | | | | | | | |  | |  |  |  |  |  |
| **8** | |  | Monthly income (Congolese Francs):……………………………………….. | | | | | | | | | | | | | |  |  |  |  |  |  |  |
| **9** | |  | Number of persons with diabetes in the household:…………………. | | | | | | | | | | | | | |  |  |  |  |  |  |  |
|  | |  | **Health system characteristics** | | | | | | | | | | | | | |  |  |  |  |  |  |  |
| **10** | |  | Distance from home to health center | | | | | | | | | | | | | |  |  |  |  |  |  |  |
|  | |  | 1. ˂ 5 Kilometers or ˂ one hour to cover | | | | | | | | | | | | | |  |  |  |  |  |  |  |
|  | |  | 2. ≥ 5 kilometers or ≥ one hour | | | | | | | | | | | | | |  |  |  |  |  |  |  |
| **11** | |  | Health insurance | | | | | | | | | | | | | |  |  |  |  |  |  |  |
|  | | | | 1. No | |  | | |  | | | | | | | | | | | | |  |  |
|  | | | | 2. Yes | |  | | |  | | | | | | | | | | | | |  |  |
|  | | | | **11a** If Yes, Specify ………. | | | |  |  | | | | | | | | | | | | |  |  |
|  | |  | **Diabetes disease and care characteristics** | | | | | | | | | | | | | |  |  |  |  |  |  |  |
|  | |  | **History** | | |  | | | | |  | | | | | |  |  |  |  |  |  |  |
| **12** | |  | Duration of Diabetes: Months | | |  | | | | |  | | | | | |  |  |  |  |  |  |  |
| **13** | |  | Comorbidity/complications : | | | | | | | |  | | | | | |  |  |  |  |  |  |  |
|  | |  | 1. Hypertension | | |  | | | | |  | | | | | |  |  |  |  |  |  |  |
|  | |  | 1. Hyperlipidemia | | |  | | | | |  | | | | | |  |  |  |  |  |  |  |
|  | |  | 1. Obesity 2. Cataracts | | |  | | | | |  | | | | | |  |  |  |  |  |  |  |
|  | |  | 1. Diabetic retinopathy | | |  | | | | |  | | | | | |  |  |  |  |  |  |  |
|  | |  | 1. Impaired renal function | | |  | | | | |  | | | | | |  |  |  |  |  |  |  |
|  | |  | 1. Erectile dysfunction | | |  | | | | |  | | | | | |  |  |  |  |  |  |  |
|  | |  | 1. Other   **13a** If Other, Specify………… | | |  | | | | |  | | | | | |  |  |  |  |  |  |  |
| **14** | |  | Hospitalizations in the last year : | | | |  | | | |  | | | | | |  |  |  |  |  |  |  |
|  | |  | 1. No | | |  | | | | |  | | | | | |  |  |  |  |  |  |  |
|  | |  | 1. Yes | | |  | | | | |  | | | | | |  |  |  |  |  |  |  |
|  | |  | **14a** If Yes, Specify below: | | |  | | | | |  | | | | | |  |  |  |  |  |  |  |
|  | **14a** | | **Number** | **Period** | | Reason | | | | | | | | | | |  |  |  |  |  |  |  |
|  | |  |  |  | |  | | | | | | | | | | |  |  |  |  |  |  |  |
|  | |  |  |  | |  | | | | | | | | | | |  |  |  |  |  |  |  |
|  | |  |  |  | |  | | | | | | | | | | |  |  |  |  |  |  |  |
|  | |  |  |  | |  | | | | | | | | | | |  |  |  |  |  |  |  |
|  | |  |  |  | |  | | | | | | | | | | |  |  |  |  |  |  |  |
|  | |  | **Lifestyle** | | |  | | | | |  | | | | | |  |  |  |  |  |  |  |
| **15** | |  | **Alcohol use/AUDIT-C** | | |  | | | | |  | | | | | |  |  |  |  |  |  |  |
|  | |  |  | | | | | | | | | | | | | |  |  |  |  |  |  |  |
|  | |  | Q1: How often did you have a drink containing alcohol in the past year? | | | | | | | | | | | | | |  |  |  |  |  |  |  |
|  | |  | Answer | | | Points | | | | | | | | | | |  |  |  |  |  |  |  |
|  | |  | Never | | | 0 | | | | | | | | | | |  |  |  |  |  |  |  |
|  | |  | Monthly or less | | | 1 | | | | | | | | | | |  |  |  |  |  |  |  |
|  | |  | Two to four times a month | | | 2 | | | | | | | | | | |  |  |  |  |  |  |  |
|  | |  | Two to three times a week | | | 3 | | | | | | | | | | |  |  |  |  |  |  |  |
|  | |  | Four or more times a week | | | 4 | | | | | | | | | | |  |  |  |  |  |  |  |
|  | |  | Q2: How many drinks did you have on a typical day when you were drinking in the past year? | | | | | | | | | | | | | |  |  |  |  |  |  |  |
|  | |  | Answer | | | Points | | | | | | | | | | |  |  |  |  |  |  |  |
|  | |  | None, I do not drink | | | 0 | | | | | | | | | | |  |  |  |  |  |  |  |
|  | |  | 1 or 2 | | | 0 | | | | | | | | | | |  |  |  |  |  |  |  |
|  | |  | 3 or 4 | | | 1 | | | | | | | | | | |  |  |  |  |  |  |  |
|  | |  | 5 or 6 | | | 2 | | | | | | | | | | |  |  |  |  |  |  |  |
|  | |  | 7 to 9 | | | 3 | | | | | | | | | | |  |  |  |  |  |  |  |
|  | |  | 10 or more | | | 4 | | | | | | | | | | |  |  |  |  |  |  |  |
|  | |  | Q3: How often did you have six or more drinks on one occasion in the past year? | | | | | | | | | | | | | |  |  |  |  |  |  |  |
|  | |  | Answer | | | Points | | | | | | | | | | |  |  |  |  |  |  |  |
|  | |  | Never | | | 0 | | | | | | | | | | |  |  |  |  |  |  |  |
|  | |  | Less than monthly | | | 1 | | | | | | | | | | |  |  |  |  |  |  |  |
|  | |  | Monthly | | | 2 | | | | | | | | | | |  |  |  |  |  |  |  |
|  | |  | Weekly | | | 3 | | | | | | | | | | |  |  |  |  |  |  |  |
|  | |  | Daily or almost daily | | | 4 | | | | | | | | | | |  |  |  |  |  |  |  |
|  | |  | **TOTAL** | | |  | | | | | | | | | | |  |  |  |  |  |  |  |
|  | |  |  | | |  | | | | |  | | | | | |  |  |  |  |  |  |  |
| **16** | | | **Smoking** | | |  | | | | |  | | | | | |  |  |  |  |  |  |  |
|  | | | Do you currently smoke cigarettes? | | | | | | | | | | | | | |  |  |  |  |  |  |  |
|  | | | 1. No | | | | | | | | | | | | | |  |  |  |  |  |  |  |
|  | | | 2. Yes | | | | | | | | | | | | | |  |  |  |  |  |  |  |
|  | | | **16a** If Yes, respond to the questions below | | | | | | | | | | | | | |  |  |  |  |  |  |  |
| **16a** | | | **Fagerstrom test for nicotine dependence** | | | | | | | | | | | | | |  |  |  |  |  |  |  |
|  | | | How soon after walking do you smoke your first cigarette? | | | Within 5 minutes | | | | |  | | | 3 | | |  |  |  |  |  |  |  |
|  |  |  |  | | | 5-30 minutes | | | | |  | | | 2 | | |  |  |  |  |  |  |  |
|  |  |  |  | | | 31-60 minutes | | | | |  | | | 1 | | |  |  |  |  |  |  |  |
|  |  |  | Do you find it difficult to refrain from smoking in places where it is forbidden? e.g. Church , Library, etc. | | | Yes | | | | |  | | | 1 | | |  |  |  |  |  |  |  |
|  |  |  |  |  |  | No | | | | |  | | | 0 | | |  |  |  |  |  |  |  |
|  |  |  | Which cigarette would you hate to give up? | | | The first in the morning | | | | |  | | | 1 | | |  |  |  |  |  |  |  |
|  |  |  |  |  |  | Any other | | | | |  | | | 0 | | |  |  |  |  |  |  |  |
|  |  |  | How many cigarettes a day do you smoke? | | | 10 or less | | | | |  | | | 0 | | |  |  |  |  |  |  |  |
|  |  |  |  |  |  | 11-20 | | | | |  | | | 1 | | |  |  |  |  |  |  |  |
|  |  |  |  |  |  | 21-30 | | | | |  | | | 2 | | |  |  |  |  |  |  |  |
|  |  |  |  |  |  | 31 or more | | | | |  | | | 3 | | |  |  |  |  |  |  |  |
|  |  |  | Do you smoke more frequently in the morning$?$ | | | Yes | | | | |  | | | 1 | | |  |  |  |  |  |  |  |
|  |  |  |  |  |  | No | | | | |  | | | 0 | | |  |  |  |  |  |  |  |
|  |  |  | Do you smoke even if you are sick in bed most of the day? | | | Yes | | | | |  | | | 1 | | |  |  |  |  |  |  |  |
|  |  |  |  | | | No | | | | |  | | | 2 | | |  |  |  |  |  |  |  |
|  |  |  | **Total score** | | | | | | | |  | | | | | |  |  |  |  |  |  |  |
|  | | |  | | | | | | | |  | | | | | |  |  |  |  |  |  |  |
| **17** | | | **Global physical activity questionnaire** | | | | | | | |  | | | | | |  |  |  |  |  |  |  |
| \| **Physical Activity** \| \| \| \| \| \| \| \| \| \| \| \| --- \| --- \| --- \| --- \| --- \| --- \| --- \| --- \| --- \| --- \| --- \| \| Next I am going to ask you about the time you spend doing different types of physical activity in a typical week. Please answer these questions even if you do not consider yourself to be a physically active person.  Think first about the time you spend doing work. Think of work as the things that you have to do such as paid or unpaid work, study/training, household chores, harvesting food/crops, fishing or hunting for food, seeking employment. *[Insert other examples if needed].* In answering the following questions 'vigorous-intensity activities' are activities that require hard physical effort and cause large increases in breathing or heart rate, 'moderate-intensity activities' are activities that require moderate physical effort and cause small increases in breathing or heart rate. \| \| \| \| \| \| \| \| \| \| \| \| **Questions** \| \| **Response** \| \| \| \| \| \| \| **Code** \| \| \| **Activity at work** \| \| \| \| \| \| \| \| \| \| \| \| 1 \| Does your work involve vigorous-intensity activity that causes large increases in breathing or heart rate like *[carrying or lifting* *heavy loads, digging or construction work*] for at least 10 minutes continuously?  *[INSERT EXAMPLES] (USE SHOWCARD)* \| \| Yes \| \| \| \| 1 \| P1 \| \| \| \| No \| \| \| \| 2  *If No, go to P 4* \| \| 2 \| In a typical week, on how many days do you do vigorous-intensity activities as part of your work? \| \| Number of days \| \| \| \| └─┘ \| P2 \| \| \| \| 3 \| How much time do you spend doing vigorous-intensity activities at work on a typical day? \| \| Hours : minutes \| \| \| \| └─┴─┘: └─┴─┘  hrs mins \| P3  (a-b) \| \| \| \| 4 \| Does your work involve moderate-intensity activity that causes small increases in breathing or heart rate such as brisk walking *[or carrying light loads*] for at least 10 minutes continuously?  *[INSERT EXAMPLES] (USE SHOWCARD)* \| \| Yes \| \| \| 1 \| \| P4 \| \| \| \| No \| \| \| 2 *If No, go to P 7* \| \| \| 5 \| In a typical week, on how many days do you do moderate-intensity activities as part of your work? \| \| Number of days \| \| \| └─┘ \| \| P5 \| \| \| \| 6 \| How much time do you spend doing moderate-intensity activities at work on a typical day? \| \| Hours : minutes \| \| \| └─┴─┘: └─┴─┘  hrs mins \| \| P6  (a-b) \| \| \| \| **Travel to and from places** \| \| \| \| \| \| \| \| \| \| \| \| The next questions exclude the physical activities at work that you have already mentioned.  Now I would like to ask you about the usual way you travel to and from places. For example to work, for shopping, to market, to place of worship. [insert other examples if needed] \| \| \| \| \| \| \| \| \| \| \| \| 7 \| Do you walk or use a bicycle (*pedal cycle*) for at least 10 minutes continuously to get to and from places? \| \| Yes \| 1 \| \| \| \| \| \| P7 \| \| No \| 2  *If No, go to P 10* \| \| \| \| \| \| \| 8 \| In a typical week, on how many days do you walk or bicycle for at least 10 minutes continuously to get to and from places? \| \| Number of days \| └─┘ \| \| \| \| \| \| P8 \| \| 9 \| How much time do you spend walking or bicycling for travel on a typical day? \| \| Hours : minutes \| └─┴─┘: └─┴─┘  hrs mins \| \| \| \| \| \| P9  (a-b) \| \| **Recreational activities** \| \| \| \| \| \| \| \| \| \| \| \| The next questions exclude the work and transport activities that you have already mentioned.  Now I would like to ask you about sports, fitness and recreational activities (leisure), [insert relevant terms]. \| \| \| \| \| \| \| \| \| \| \| \| 10 \| Do you do any vigorous-intensity sports, fitness or recreational (*leisure*) activities that cause large increases in breathing or heart rate like [*running or football,]* for at least 10 minutes continuously?  *[INSERT EXAMPLES] (USE SHOWCARD)* \| \| Yes \| \| 1 \| \| \| P10 \| \| \| \| No \| \| 2  *If No, go to P 13* \| \| \| \| 11 \| In a typical week, on how many days do you do vigorous-intensity sports, fitness or recreational (*leisure*) activities? \| \| Number of days \| \| └─┘ \| \| \| P11 \| \| \| \| 12 \| How much time do you spend doing vigorous-intensity sports, fitness or recreational activities on a typical day? \| \| Hours : minutes \| \| └─┴─┘: └─┴─┘  hrs mins \| \| \| P12  (a-b) \| \| \| \| 13 \| Do you do any moderate-intensity sports, fitness or recreational *(leisure*) activities that causes a small increase in breathing or heart rate such as brisk walking*,*(*cycling, swimming, volleyball*)for at least 10 minutes continuously?  *[INSERT EXAMPLES] (USE SHOWCARD)* \| \| Yes \| \| 1 \| \| \| P13 \| \| \| \| No \| \| 2  *If No, go to P16* \| \| \|  \| \| \| \| 14 \| In a typical week, on how many days do you do moderate-intensity sports, fitness or recreational (*leisure*) activities? \| \| Number of days \| \| └─┘ \| \| \| P14 \| \| \| \| 15 \| How much time do you spend doing moderate-intensity sports, fitness or recreational (*leisure*) activities on a typical day? \| \| Hours : minutes \| \| └─┴─┘: └─┴─┘  hrs mins \| \| \| P15  (a-b) \| \| \| \| **Sedentary behaviour** \| \| \| \| \| \| \| \| \| \| \| \| The following question is about sitting or reclining at work, at home, getting to and from places, or with friends including time spent [sitting at a desk, sitting with friends, travelling in car, bus, train, reading, playing cards or watching television], but do not include time spent sleeping.  *[INSERT EXAMPLES] (USE SHOWCARD)* \| \| \| \| \| \| \| \| \| \| \| \| 16 \| How much time do you usually spend sitting or reclining on a typical day? \| \| Hours : minutes \| \| └─┴─┘: └─┴─┘  hrs mins \| \| \| P16  (a-b) \| \| \| \|  \| **GLOBAL PHYSICAL ACTIVITY SCORE** \| \|  \| \| \| \| \| \| \| \| | | | | | | | | | | | | | | | | |  |  |  |  |  |  |  |
|  | |  | **Clinical parameters** | | |  | | | | |  | | |  | | |  | |  |  |  |  |  |
| **18** | |  | Weight (Kg): ……………… | | |  | | | | |  | | |  | | |  | |  |  |  |  |  |
| **19** | |  | Height (centimeters): …… | | |  | | | | |  | | |  | | |  | |  |  |  |  |  |
| **20** | |  | BMI (Kg/m2):…………………… | | |  | | | | |  | | |  | | |  | |  |  |  |  |  |
| **21** | |  | Waist circumference: ……..cms | | |  | | | | |  | | |  | | |  | |  |  |  |  |  |
| **22** | |  | Blood pressure: …../…….mmHg | | |  | | | | |  | | |  | | |  | |  |  |  |  |  |
|  | |  | **Treatment** | | |  | | | | |  | | |  | | |  | |  |  |  |  |  |
|  | |  | **Regimen**  **23a** Insulin: Type:………….. | | |  | | | | |  | | |  | | |  | |  |  |  |  |  |
|  | |  | **23b** Oral Antidiabetic Drugs:………….. | | |  | | | | |  | | |  | | |  | |  |  |  |  |  |
|  | |  | 1. Single:……… | | |  | | | | |  | | |  | | |  | |  |  |  |  |  |
|  | |  | 2. Mixed: ……….. | | |  | | | | |  | | |  | | |  | |  |  |  |  |  |
|  | |  | **23c** Mixed Insulin-Oral Antidiabetic Drugs: ….. | | | | | | | | |  | | |  |  |  | |  |  |  |  |  |
|  | |  | **23d** Other:………………….  **Total duration of treatment**  **23e** Number of months:…………………… | | |  | | | | |  | | |  | | |  | |  |  |  |  |  |

| **Patient health questionnaire-9 (PHQ-9)** | | | | | | | | | | | | | |
| --- | --- | --- | --- | --- | --- | --- | --- | --- | --- | --- | --- | --- | --- |
| Over the last 2 weeks, how often have you been bothered by any of the following problems? | | | | | | | | | | | | | |
|  | | | | | | | | | | | | | |
|  |  | | | | Not at all | | | Several days | More than half the days | | | Nearly every day | |
|  |  | | | | 0 | | | 1 | 2 | | | 3 | |
| 1 | Little interest or pleasure in doing things | | | | 0 | | | 1 | 2 | | | 3 | |
| 2 | Feeling down, depressed, or hopeless | | | | 0 | | | 1 | 2 | | | 3 | |
| 3 | Trouble failing or staying asleep, or sleeping too much | | | | 0 | | | 1 | 2 | | | 3 | |
| 4 | Feeling tired or having little energy | | | | 0 | | | 1 | 2 | | | 3 | |
| 5 | Poor appetite or overeating | | | | 0 | | | 1 | 2 | | | 3 | |
| 6 | Feeling bad about yourself-or that you are a failure or have let yourself or your family down | | | | 0 | | | 1 | 2 | | | 3 | |
| 7 | Trouble concentrating on things, such as reading the newspaper or watching television | | | | 0 | | | 1 | 2 | | | 3 | |
| 8 | Moving or speaking so slowly that other people could have noticed. Or the opposite-being so figety or restless that you have been moving around a lot more than usual | | | | 0 | | | 1 | 2 | | | 3 | |
| 9 | Thoughts that you would be better off dead, or of hurting yourself | | | | 0 | | | 1 | 2 | | | 3 | |
|  |  | | | | Add columns | | |  |  | | |  | |
| **24** | | | | | Total | | |  | | | | | |
| **Diabetes distress study (DDS)** | | | | | | | | | | | | |  |
| Living with diabetes can sometimes be tough. There may be many problems and hassles concerning diabetes and they can vary greatly in severity. Problems may range from minor hassles to major life difficulties. Listed below are 17 potential problem areas that people with diabetes may experience. Consider the degree to which each of the 17 items may have distressed or bothered you DURING THE PAST MONTH. | | | | | | | | | | | | |  |
| Please note that we are asking you to indicate the degree to which each item may be bothering you in your life, NOT whether the item is merely true for you. If you feel that a particular item is not a bother or a problem for you, we would circle "1". If it is very bothersome to you, we might circle "6". | | | | | | | | | | | | |  |
|  | | | Not a problem | A slight problem | | A moderate problem | Somewhat serious problem | | | A serious problem | A very serious problem | |  |
| 1. Feeling that diabetes is taking up too much of my mental and physical energy every day | | | 1 | 2 | | 3 | 4 | | | 5 | 6 | |  |
| 2. Feeling that my doctor doesn't  know enough about diabetes and  diabetes care. | | | 1 | 2 | | 3 | 4 | | | 5 | 6 | |  |
| 3. Not feeling confident in my  day-to-day ability to manage  diabetes. | | | 1 | 2 | | 3 | 4 | | | 5 | 6 | |  |
| 4. Feeling angry, scared and/or  depressed when I think about  living with diabetes. | | | 1 | 2 | | 3 | 4 | | | 5 | 6 | |  |
| 5. Feeling that my doctor doesn't  give me clear enough directions on how to manage my diabetes. | | | 1 | 2 | | 3 | 4 | | | 5 | 6 | |  |
| 6. Feeling that I am not testing my  blood sugars frequently enough. | | | 1 | 2 | | 3 | 4 | | | 5 | 6 | |  |
| 7. Feeling that I will end up with  serious long-term complications,  no matter what I do. | | | 1 | 2 | | 3 | 4 | | | 5 | 6 | |  |
| 8. Feeling that I am often failing  with my diabetes routine. | | | 1 | 2 | | 3 | 4 | | | 5 | 6 | |  |
| 9. Feeling that friends or family  are not supportive enough of  self-care efforts (e.g. planning  activities that conflict with my  schedule, encouraging me to  eat the "wrong" foods). | | | 1 | 2 | | 3 | 4 | | | 5 | 6 | |  |
| 10. Feeling that diabetes controls  my life. | | | 1 | 2 | | 3 | 4 | | | 5 | 6 | |  |
| 11. Feeling that my doctor doesn't  take my concerns seriously  enough. | | | 1 | 2 | | 3 | 4 | | | 5 | 6 | |  |
| 12. Feeling that I am not sticking  closely enough to a good meal  plan. | | | 1 | 2 | | 3 | 4 | | | 5 | 6 | |  |
| 13. Feeling that friends or family  don't appreciate how difficult  living with diabetes can be. | | | 1 | 2 | | 3 | 4 | | | 5 | 6 | |  |
| 14. Feeling overwhelmed by the  demands of living with diabetes. | | | 1 | 2 | | 3 | 4 | | | 5 | 6 | |  |
| 15. Feeling that I don't have a  doctor who I can see regularly  enough about my diabetes. | | | 1 | 2 | | 3 | 4 | | | 5 | 6 | |  |
| 16. Not feeling motivated to keep  up my diabetes self-management. | | | 1 | 2 | | 3 | 4 | | | 5 | 6 | |  |
| 17. Feeling that friends or family  don't give me the emotional  support that I would like. | | | 1 | 2 | | 3 | 4 | | | 5 | 6 | |  |
| **25** | | **25a** | Total score | | | | | | | |  | |  |
|  | | **25b** | Emotional burden | | | | | | | |  | |  |
|  | | **25c** | Physician burden | | | | | | | |  | |  |
|  | | **25d** | Regimen burden | | | | | | | |  | |  |
|  | | **25e** | Interpersonal burden | | | | | | | |  | |  |

| **Diabetes self-management questionnaire (DSMQ)** | | | | | | | |
| --- | --- | --- | --- | --- | --- | --- | --- |
|  | | | | | | | |
| The following statements describe self-care activities related to your diabetes. Thinking about your self-care over the **last 8 weeks**, please specify the extent to which each statement applies to you.  Note: If you monitor your glucose using continuous interstitial glucose monitoring (CGM), please refer to this where ‘blood sugar checking’ is requested. | | | | applies to me very much | applies to me to a consider-able degree | applies to me to some degree | does not apply to me |
| 1. | I check my blood sugar levels with care and attention.  *Blood sugar measurement is not required as a part of my treatment.* | | | 3 | 2 | 1 | 0 |
| 2. | The food I choose to eat makes it easy to achieve optimal blood sugar levels. | | | 3 | 2 | 1 | 0 |
| 3. | I keep all doctors’ appointments recommended for my diabetes treatment. | | | 3 | 2 | 1 | 0 |
| 4. | I take my diabetes medication (e. g. insulin, tablets) as prescribed.  *Diabetes medication/insulin is not required as a part of my treatment.* | | | 3 | 2 | 1 | 0 |
| 5. | Occasionally I eat lots of sweets or other foods rich in carbohydrates. | | | 3 | 2 | 1 | 0 |
| 6. | I record my blood sugar levels regularly (or analyse the value chart with my blood glucose meter).  *Blood sugar measurement is not required as a part of my treatment.* | | | 3 | 2 | 1 | 0 |
| 7. | I tend to avoid diabetes-related doctors’ appointments. | | | 3 | 2 | 1 | 0 |
| 8. | I do regular physical activity to achieve optimal blood sugar levels. | | | 3 | 2 | 1 | 0 |
| 9. | I strictly follow the dietary recommendations given by my doctor or diabetes specialist. | | | 3 | 2 | 1 | 0 |
| 10. | I do not check my blood sugar levels frequently enough as would be required for achieving good blood glucose control.  *Blood sugar measurement is not required as a part of my treatment.* | | | 3 | 2 | 1 | 0 |
| 11. | I avoid physical activity, although it would improve my diabetes. | | | 3 | 2 | 1 | 0 |
| 12. | I tend to forget to take or skip my diabetes medication (e. g. insulin, tablets).  *Diabetes medication/insulin is not required as a part of my treatment.* | | | 3 | 2 | 1 | 0 |
| 13. | Sometimes I have real ‘food binges’ (not triggered by hypoglycaemia). | | | 3 | 2 | 1 | 0 |
| 14. | Regarding my diabetes care, I should see my medical practitioner(s) more often. | | | 3 | 2 | 1 | 0 |
| 15. | I tend to skip planned physical activity. | | | 3 | 2 | 1 | 0 |
| 16. | My diabetes self-care is poor. | | | 3 | 2 | 1 | 0 |
| **26** | | **26a** | Total score |  | | | |
|  | | **26b** | Dietary control |  | | | |
|  | | **26c** | Glucose management |  | | | |
|  | | **26d** | Physical activity |  | | | |
|  | | **26e** | Physician contact |  | | | |

| **Household food insecurity access scale(HFIAS)** | | | |
| --- | --- | --- | --- |
|  | | | |
| **No** | **Question** | **Response Options** | **Code** |
| 1 | In the past four weeks, did you worry that your household would not have enough food? | 0 = No (skip to Q2)  1=Yes | \| ….\|___\| \| \| --- \| |
| 1.a | How often did this happen? | 1 = Rarely (once or twice in the past four weeks)  2 = Sometimes (three to ten times in the past four weeks)  3 = Often (more than ten times in the past four weeks) | \| ….\|___\| \| \| --- \| |
| 2. | In the past four weeks, were you or any household member not able to eat the kinds of foods you preferred because of a lack of resources? | 0 = No (skip to Q3)  1=Yes | \| ….\|___\| \| \| --- \| |
| 2.a | How often did this happen? | 1 = Rarely (once or twice in the past four weeks)  2 = Sometimes (three to ten times in the past four weeks)  3 = Often (more than ten times in the past four weeks) | \| ….\|___\| \| \| --- \| |
| 3. | In the past four weeks, did you or any household member have to eat a limited variety of foods due to a lack of resources? | 0 = No (skip to Q4)  1=Yes | \| ….\|___\| \| \| --- \| |
| 3.a | How often did this happen? | 1 = Rarely (once or twice in the past four weeks)  2 = Sometimes (three to ten times in the past four weeks)  3 = Often (more than ten times in the past four weeks) | \| ….\|___\| \| \| --- \| |
| 4. | In the past four weeks, did you or any household member have to eat some foods that you really did not want to eat because of a lack of resources  to obtain other types of food? | 0 = No (skip to Q5)  1=Yes | \| ….\|___\| \| \| --- \| |
| 4.a | How often did this happen? | 1 = Rarely (once or twice in the past four weeks)  2 = Sometimes (three to ten times in the past four weeks)  3 = Often (more than ten times in the past four weeks) | \| ….\|___\| \| \| --- \| |
| 5. | In the past four weeks, did you or any household member have to eat a smaller meal than you felt you needed because there was not enough food? | 0 = No (skip to Q6)  1=Yes | \| ….\|___\| \| \| --- \| |
| 5.a | How often did this happen? | 1 = Rarely (once or twice in the past four weeks)  2 = Sometimes (three to ten times in the past four weeks)  3 = Often (more than ten times in the past four weeks) | \| ….\|___\| \| \| --- \| |
| 6. | In the past four weeks, did you or any other household member have to eat fewer meals in a day because there was not enough food? | 0 = No (skip to Q7)  1=Yes | \| ….\|___\| \| \| --- \| |
| 6.a | How often did this happen? | 1 = Rarely (once or twice in the past four weeks)  2 = Sometimes (three to ten times in the past four weeks)  3 = Often (more than ten times in the past four weeks) | \| ….\|___\| \| \| --- \| |
| 7. | In the past four weeks, was there ever no food to eat of any kind in your household because of lack of resources to get food? | 0 = No (skip to Q8)  1=Yes | \| ….\|___\| \| \| --- \| |
| 7.a | How often did this happen? | 1 = Rarely (once or twice in the past four weeks)  2 = Sometimes (three to ten times in the past four weeks)  3 = Often (more than ten times in the past four weeks) | \| ….\|___\| \| \| --- \| |
| 8. | In the past four weeks, did you or any household member go to sleep at night hungry because there was not enough food? | 0 = No (skip to Q9)  1=Yes | \| ….\|___\| \| \| --- \| |
| 8.a | How often did this happen? | 1 = Rarely (once or twice in the past four weeks)  2 = Sometimes (three to ten times in the past four weeks)  3 = Often (more than ten times in the past four weeks) | \| ….\|___\| \| \| --- \| |
| 9. | In the past four weeks, did you or any household member go a whole day and night without eating anything because there was not enough food? | 0 = No (questionnaire is finished)  1=Yes | \| ….\|___\| \| \| --- \| |
| 9.a | How often did this happen? | 1 = Rarely (once or twice in the past four weeks)  2 = Sometimes (three to ten times in the past four weeks)  3 = Often (more than ten times in the past four weeks) | \| ….\|___\| \| \| --- \| |
| **27** |  | HFIAS category |  |

| **Revised Michigan knowledge questionnaire** | | | |
| --- | --- | --- | --- |
| Here are 20 statements about diabetes, some are true statements and some are false. Please read each statement and then indicate whether you think it is true or false or you don’t know. | | | |
| 1 | The diabetes diet is a healthy diet for most people | | TRUE / FALSE / DON’T KNOW |
| 2 | Glycosylated haemoglobin (HbA1c) is a test that measures your average blood glucose level in the past week. | | TRUE / FALSE / DON’T KNOW |
| 3 | A pound of chicken has more carbohydrate in it than a pound of potatoes. | | TRUE / FALSE / DON’T KNOW |
| 4 | Orange juice has more fat in it than low fat milk. | | TRUE / FALSE / DON’T KNOW |
| 5 | Urine testing and blood testing are both equally as good for testing the level of blood glucose. | | TRUE / FALSE / DON’T KNOW |
| 6 | Unsweetened fruit juice raises blood glucose levels. | | TRUE / FALSE / DON’T KNOW |
| 7 | A can of diet soft drink can be used for treating low blood glucose levels. | | TRUE / FALSE / DON’T KNOW |
| 8 | Using olive oil in cooking can help lower the cholesterol in your blood. | | TRUE / FALSE / DON’T KNOW |
| 9 | Exercising regularly can help reduce high blood pressure. | | TRUE / FALSE / DON’T KNOW |
| 10 | For a person in good control, exercising has no effect on blood sugar levels. | | TRUE / FALSE / DON’T KNOW |
| 11 | Infection is likely to cause an increase in blood sugar levels. | | TRUE / FALSE / DON’T KNOW |
| 12 | Wearing shoes a size bigger than usual helps prevent foot ulcers. | | TRUE / FALSE / DON’T KNOW |
| 13 | Eating foods lower in fat decreases your risk for heart disease. | | TRUE / FALSE / DON’T KNOW |
| 14 | Numbness and tingling may be symptoms of nerve disease. | | TRUE / FALSE / DON’T KNOW |
| 15 | Lung problems are usually associated with having diabetes. | | TRUE / FALSE / DON’T KNOW |
| 16 | When you are sick with the flu you should test for glucose more often. | | TRUE / FALSE / DON’T KNOW |
| **SKIP TO QUESTION 19 IF YOU DON’T TAKE INSULIN** | | | |
| 17 | High blood glucose levels may be caused by too much insulin. | | TRUE / FALSE / DON’T KNOW |
| 18 | If you take your morning insulin but skip breakfast your blood glucose level will usually decrease. | | TRUE / FALSE / DON’T KNOW |
| 19 | Having regular check-ups with your doctor can help spot the early signs of diabetes complications. | | TRUE / FALSE / DON’T KNOW |
| 20 | Attending your diabetes appointments will stop you getting diabetes complications. | | TRUE / FALSE / DON’T KNOW |
| **28** |  | TOTAL SCORE |  |

| **MULTIDIMENSIONAL SCALE OF PERCEIVED SOCIAL SUPPORT** | | | | | | | | |
| --- | --- | --- | --- | --- | --- | --- | --- | --- |
| Instructions: We are interested in how you feel about the following statements. We will read each statement; tell us how you feel about each statement. | | | | | | | | |
| “1” is for **Very Strongly Disagree**  “2” is for **Strongly Disagree**  “3” is for **Mildly Disagree**  “4” is for **Neutral** | | | | “5” is for **Mildly Agree**  “6” is for **Strongly Agree**  “7” is for **Very Strongly Agree** | | | | |
|  |  | Very Strongly Disagree | Strongly  Disagree | Mildly Disagree | Neutral | Mildly Agree | Strongly Agree | Very Strongly Agree |
| 1 | There is a special person who is around when I am in need. | 1 | 2 | 3 | 4 | 5 | 6 | 7 |
| 2 | There is a special person with whom I can share joys and sorrows. | 1 | 2 | 3 | 4 | 5 | 6 | 7 |
| 3 | My family really tries to help me. | 1 | 2 | 3 | 4 | 5 | 6 | 7 |
| 4 | I get the emotional help & support I need from my family. | 1 | 2 | 3 | 4 | 5 | 6 | 7 |
| 5 | I have a special person who is a real source of comfort to me. | 1 | 2 | 3 | 4 | 5 | 6 | 7 |
| 6 | My friends really try to help me. | 1 | 2 | 3 | 4 | 5 | 6 | 7 |
| 7 | I can count on my friends when things go wrong. | 1 | 2 | 3 | 4 | 5 | 6 | 7 |
| 8 | I can talk about my problems with my family. | 1 | 2 | 3 | 4 | 5 | 6 | 7 |
| 9 | I have friends with whom I can share my joys and sorrows. | 1 | 2 | 3 | 4 | 5 | 6 | 7 |
| 10 | There is a special person in my life who cares about my feelings. | 1 | 2 | 3 | 4 | 5 | 6 | 7 |
| 11 | My family is willing to help me make decisions. | 1 | 2 | 3 | 4 | 5 | 6 | 7 |
| 12 | I can talk about my problems with my friends. | 1 | 2 | 3 | 4 | 5 | 6 | 7 |
| **29** |  | | **29a** | Significant other | |  | | |
|  |  | | **29b** | Family | |  | | |
|  |  | | **29c** | Friends | |  | | |
|  |  | | **29d** | Total | |  | | |

| **MORISKY GREEN LEVINE TEST** | | | |
| --- | --- | --- | --- |
|  | Yes | No |  |
| Do you ever forget to take your medicine? | |  |  |
| Are you careless at times about taking your medicine? | |  |  |
| When you feel better do you sometimes stop taking your medicine? | |  |  |
| Sometimes you feel worse, when you take the medicine, do you stop taking it? | |  |  |
| **30** | | **MGL Category** |  |

**THANK YOU FOR YOUR PARTICIPATION**
