# Supplementary material for: Protocol: Developing a framework to improve glycaemic control among patients with type 2 diabetes mellitus in Kinshasa, Democratic Republic of the Congo
Source: PLoS One. 2022 Sep 26;17(9):e0268177. doi: 10.1371/journal.pone.0268177 (PMC9512168; doi:10.1371/journal.pone.0268177)
Supplement: S3 Appendix — (DOCX) [file pone.0268177.s003.docx]

| **STUDY TITLE:** DEVELOPING A FRAMEWORK TO IMPROVE GLYCAEMIC CONTROL AMONG PATIENTS WITH TYPE 2 DIABETES MELLITUS IN KINSHASA, DEMOCRATIC REPUBLIC OF THE CONGO  **S3 APPENDIX: LABORATORY SHEET** |
| --- |
| **ID:……………………………… Province:……………………** |
| **Place:………………………..... Investigator number:…………**  **Date: / /** |

|  | **Laboratory measurements** |  |
| --- | --- | --- |
|  | HbA1C: …………………..% |  |
