# Supplementary material for: Protocol: Developing a framework to improve glycaemic control among patients with type 2 diabetes mellitus in Kinshasa, Democratic Republic of the Congo
Source: PLoS One. 2022 Sep 26;17(9):e0268177. doi: 10.1371/journal.pone.0268177 (PMC9512168; doi:10.1371/journal.pone.0268177)
Supplement: S4 Appendix — (DOCX) [file pone.0268177.s004.docx]

**STUDY TITLE:** DEVELOPING A FRAMEWORK TO IMPROVE GLYCAEMIC CONTROL AMONG PATIENTS WITH TYPE 2 DIABETES MELLITUS IN KINSHASA, DEMOCRATIC REPUBLIC OF THE CONGO

**S4 APPENDIX: INTERVIEW GUIDE FOR IN-DEPTH INTERVIEW**

**FOR HEALTHCARE PROVIDERS**

**Topic I: Organization and equipment for diabetes care**

Can you describe the organization of your healthcare structure for diabetes (human resource, financement of care…)?

What are the failures of this organization in regard to diabetes care?

How do you think your structure must be organized for better care?

What equipment you need the most for diabetes care and follow-up?

What are the documents do you use for diabetes care (guidelines, protocols….)?

How is feasible the support from other categories of health providers in your practice?

**Topic II: Support to self-management and prevention**

What is your understanding of self-management of diabetes?

How do you practically approach your patients to support self-management? How are you encouraging patient involvement in the care (treatment…)?

How would your healthcare system be improved by transferring activities for diabetes treatment and follow-up to patients?

How are you organizing feedbacks to patients in regard to treatment follow-up?

What are the barriers have you identified to effectively support self-management of diabetes?

How are you determining therapeutic goals for patients?

How are you identifying high risk patients for poor glycaemic control? How do you approach these patients?

What are the most important skills you show to patients to avoid complications?

What are resources available for reaching therapeutic goals?

Tell us about your motivation in taking care of patients with type 2 diabetes

**Topic III: Information systems**

How is important for you to report your activities?

How is your organization organizing the referral?

How are you identifying patients with special needs or at risk?

How are you organized to remind patients about their follow up or care?

How to better report the impact of diabetes in the healthcare system?

**Topic IV: Continuity and coordination of care**

How do you collaborate with other caregivers of your patients?

What are the documents used for this purpose?

Who is acting as coordinator of care for your patients?

**Topic V: Policy environment**

What is your basic education? What is the training received to take care of patients with diabetes? How are organize continual professional development activities?

**Topic VI: Closing the interview**

Additional comments

Acknowledgements

**FOR PATIENTS WITH DIABETES**

**Topic I: Self-management**

How do you manage to maintain control of diabetes mellitus?

What resources do you think can be useful to deal with challenges in diabetes?

What kind of support from the healthcare providers you expect for better care?

Do you have any ideas as to changes in the management of diabetes mellitus?

How would you prevent complications?

**Topic II: Healthcare system**

Can you critically appraise the care you receive from your healthcare facility and indicate what are changes would you recommend?

How would you appreciate feedback from your healthcare givers?

**Topic III: Policy environment**

What would be your expectations for what the decision-makers can do for great impact on diabetes care?

How can your communication with the healthcare provider could be improved? How can your access to care (geographical, financial…) be improved?

**Topic IV: Community**

Tell us how living with diabetes has stigma attached to it

How to deal with stigmatization about patients with diabetes in communities?

Would you appreciate any complimentary service in the community you live in? If any, can you describe the type of service?

**Topic V: Closing the interview**

Additional comments

Acknowledgements
