# Supplementary material for: Protocol: Developing a framework to improve glycaemic control among patients with type 2 diabetes mellitus in Kinshasa, Democratic Republic of the Congo
Source: PLoS One. 2022 Sep 26;17(9):e0268177. doi: 10.1371/journal.pone.0268177 (PMC9512168; doi:10.1371/journal.pone.0268177)
Supplement: Attachment — Submitted filename: Review_PONE-D-21-29300_PD.pdf [file pone.0268177.s005.pdf]

Manuscript: “Protocol: Developing a framework to improve glycaemic control among patients with type 2 diabetes mellitus in Kinshasa, Democratic Republic of the Congo”

Reviewer: Pauline Dreesen

Summary of the research

The manuscript describes the protocol of a study that will take place in Kinshasa, Democratic Republic of the Congo, and aims at defining drivers for poor glyaecemic control in type 2 diabetes patients (T2DM) and subsequently the development of an intervention package to improve the glycemic control in this population. The protocol consists of three sub-studies:
Sub-study 1 is a multicentric study (1 referral hospital and 65 health centers) consisting of two components: a qualitative and a quantitative phase. This sub-study aims to identify drivers of poor glycaemic control among type 2 diabetes patients, defined as HbA1c < 7%. The qualitative phase aims to explore the experiences and perspectives of 20 T2DM patients through in-depth interviews. The quantitative phase is a cross-sectional study including 614 patients across 20 randomly selected health facilities consisting of different questionnaires related to psychological, socio-demographic, clinical, physical, and social factors, among others, together with a HbA1c blood sampling. Next, a qualitative study will take place in 4 clinics in Kinshasa and aims at exploring T2DM patients’ and healthcare providers’ perspectives on improving glycaemic control. In-depth interviews will be carried out with at least 20 patients and 20 health care providers until thematic saturation. The last part of the study protocol consists of developing an interventions package for improving glycaemic control based on the Delphi method. 25 experts in the field of diabetes management in Kinshasa will be invited to participate in an anonymous electronic survey consisting of 3 rounds, to reach a consensus.

Strengths:

In Kinshasa, a high percentage of T2DM patients (68%-86%) have poor glycemic control, leading to an increased risk for complications and health care costs. So far, the experiences and perspectives of both diabetic patients and healthcare providers have not been considered in determining: (1) the drivers for poor glycemic control and (2) interventions for improving glycemic control. These factors are explored in this study, together with the input from stakeholders and experts in the field, which makes the study a relevant and innovative topic.

Weaknesses:

Although the manuscript recognizes the importance of restructuring health systems with greater empowerment of patients, involvement of their families and the communities in the care, the perspectives of family members are not taken into account in this study.

There is a high risk for selection biases, which have also been described in the manuscript.

Minor revisions:

Abstract

1. I would suggest presenting the numbers on T2DM patients with poor glycaemic control in Kinshasa instead of reporting ‘a large proportion of patients’.
2. I would suggest providing more details on the qualitative study as part of sub-study 1 (e.g. in-depth review).

Introduction

1. For non-expert readers in the field: could you elaborate on/provide more details or examples of the adjustments patients with diabetes are required to make, as well as on the definition of glycaemic control (this is not mentioned until the methodology part of the paper (HbA1c < 7%))?
2. 50% of patients achieve glycaemic control worldwide. Does this number also include T1DM?
3. Could you provide more details on why poor glycaemic control in T2DM patients varies across settings and are complex?
4. It is unclear whether the reported prevalence of diabetes in the DRC (4.8%) includes both T1DM and T2DM.
5. In the following sentence, it is not clear to me what 'the development' refers to: ‘ The burden of diabetes is a real hindrance to the development of both patients and their families, and also of the health system oriented towards other priorities with limited resources’

Materials and methods

Sub-study 1:

Questions related to the qualitative phase:

1. The manuscript describes the interview of 20 patients until thematic saturation is reached. Patients are selected purposively with both good and poor glycaemic control. Will a blood sampling be performed before the interview to determine the HbA1c level, or how is the glycaemic control prior to the interview defined? How is this number (20 interviews) distributed across the patients with good and poor glycaemic control?
2. Is it necessary to interview T2DM patients with good glycaemic control, since the research objective is to define drivers for poor glyaecemic control? Or is another objective to define drivers for good glycaemic control as well?
3. Is there a difference made between patient with treatment < / > 7 years (cf. quantitative phase)?
4. Are the interviews performed in 1 of the 66 centers (which one(s))?
5. How much time will the interview approximately take?

Questions related to the quantitative phase:

6. The protocol describes an interview procedure, which, in my opinion, is confusing since validated questionnaires are used.
7. Since only 20 centers are selected to recruit patients, it seems to me the multicentric study does not comprise 66 centers.

8. Is there a specific reason why the participants will not fill in the questionnaires themselves, but the researchers ask the questions? Will this not create an interpretation bias? Can the questionnaires be completed online by the participants in their home situation?
9. Why are the characteristics of diabetic disease not collected from the patient's medical file?
10. How much time will the completion of the questionnaires take? It seems a lot of information is asked (18 pages); is this all necessary information related to the research objective?

Sub-study 2

11. Patients will be selected if they have been followed for at least six months for their diabetes and if they have integrated the model of care offered to patients with diabetes. It is not clear what this 'model of care' comprises.
