# Supplementary material for: Protocol: Developing a framework to improve glycaemic control among patients with type 2 diabetes mellitus in Kinshasa, Democratic Republic of the Congo
Source: PLoS One. 2022 Sep 26;17(9):e0268177. doi: 10.1371/journal.pone.0268177 (PMC9512168; doi:10.1371/journal.pone.0268177)
Supplement: Attachment — Submitted filename: Responses to reviewers.pdf [file pone.0268177.s006.pdf]

29 March 2022

RESPONSES TO REVIEWERS' COMMENTS

Title: Protocol: Developing a framework to improve glycaemic control among patients with type 2 diabetes mellitus in Kinshasa, Democratic Republic of the Congo

Reference: PONE-D-21-29300

I thank you and the reviewers for having taken time to read the manuscript and having made valuable remarks. Your remarks have been taken in account in improving the protocol. The table below highlights the reviewers 'comments and how these have been addressed.

**Reviewers' comments**	**Responses from author**	**Document/Page**
**Journal requirements**		
We note that you have stated that you will provide repository information for your data at acceptance. Should your manuscript be accepted for publication, we will hold it until you provide the relevant accession numbers or DOIs necessary to access your data. If you wish to make changes to your Data Availability statement, please describe these changes in your cover letter and we will update your Data Availability statement to reflect the information you provide.	This is a study protocol. It describes the plan of studies that will be conducted and published subsequently until the completion of a PhD degree over four years. The data underlying the findings of the three studies will be published as attachments. A change for the Data availability statement has been mentioned in the cover letter.	Cover letter
Please amend your authorship list in your manuscript file to include the author list	The authorship in the manuscript has been amended.	Page 1
**Reviewer#1**		
Depending on the local culture, the authors may be a little ambitious to count on a rate as low as 10% for non-responders for sub-study 1.	We thank the reviewer for the valuable comment. We decided on the 10% non-response rate based on the experience from other studies on diabetes in the study setting. For example, studies by Muyer et al in 2012, and Cedrick et al in	No change

	2021 had respectively a non-response rate of 6.3% and 4.5% (Muyer *et al.*, 2012, Cedrick *et al.*, 2021).<br><br>References<br><br>Cedrick, L. M. *et al.* (2021) 'Prevalence and determinants of poor glycaemic control amongst patients with diabetes followed at Vanga Evangelical Hospital, Democratic Republic of the Congo, *African journal of primary health care & family medicine*, 13(1). doi: 10.4102/phcfm.v13i1.2664.<br><br>Muyer, M. T. *et al.* (2012) 'Diabetes and intermediate hyperglycaemia in Kisantu, DR Congo: A cross-sectional prevalence study', *BMJ Open*, 2(6), pp. 1–7. doi: 10.1136/bmjopen-2012-001911.	
**Reviewer#2**		
The abstract does not clearly separate the three aims of this study	Thanks for this comment. We have now specified the aim for each of the proposed studies.	Abstract<br>Lines 46-47, 55-57, 57-59
Line 32, conducting multivariable logistic regression is ok, but only after having identified confounding factors	Thanks for this comment. In the process described for data analysis, we have planned to conduct bivariate analysis before multivariable analysis and include variables with $p<0.20$ in the multivariable models. We also specified confounders a priori as age, sex, treatment duration, and food security.<br><br>We have updated the abstract accordingly to include the planned analyses.	Lines 51-52

Line 465 please modify the timeline to actual timing	We are grateful for this comment. The timeline has been updated.	Line 533: Table 1
Introduction line 56-57: be more specific when referring to “setting”	Thanks for this comment. We have changed ‘settings’ to ‘locations’ to be more accurate.	Line 82
Study setting line 134: any reference for the number of inhabitants?	A reference for the number of inhabitants has been included.	Line 161<br>Line 664: Reference number 26
Line 158: selection of patient for interview can induce an obvious bias. Why not invite randomly or all participants until saturation is reached?	Thanks for this comment. Given the nature of the study design, the intention is not to recruit a representative sample but patients who are information-rich and would be likely to share their perspectives on glycaemic control.	No change
Line 230: Not clear when during the interview procedure all the questionnaires will be administered: Furthermore, there is some concerns that the multiplicity of questionnaires, the time necessary for completing them and especially the fact that if it is too long the participant might answer inappropriately. How does the author address this specific issue?	Thanks for this comment. Once an eligible patient is identified and that his/her consent is obtained for the study, physical/anthropometric measurements will be taken. After that, the questionnaire is administered by a data collector.<br>At the end, the blood sample is taken.<br><br>It is estimate that the time to complete the questionnaire would be approximately up to 60 minutes.<br><br>The participants will be informed of the details of the study, issues of reimbursements and will only participate once done with other clinic activities or while waiting to be attended.	Lines 272-275<br><br><br><br><br>Line 274<br><br><br>Line 196-198
Line 237, would recommend adding the current working status	Thanks for comment. The current working status is one of the study’ variables of the	S2 Appendix, page 2, question 7

	study as it can be verified in the data collection tool.<br>It was unfortunately forgotten in the list of variables.<br><br>The list of the variables has been reviewed to include all the information sought from participants.	 <br><br><br><br><br>Line 280 and named occupation
No information is given regarding who will drive the Delphy consensus data collection and interpretation.	Thanks for this comment. A research team lead by the Principal Investigator and including at least one supervisor and a statistician will conduct data collection and interpretation of the data. The information has been added to the protocol.	Lines 449-451
No information is given regarding the research assistant's integration in the authorship.	Thanks for the comment. The research assistant will be acknowledged for their support in the study. For the authorship, we will follow guidelines from the International Committee of Medical Journal Editors (ICMJE). I added the above information in the protocol.	Lines 525-528<br><br>Reference 51
Is the retribution of the participants specifically specified before inclusion? It might obviously induce some bias	The retribution for the participants is described in the informed consent form. A reimbursement of the transport is only given when the patient is invited for interviews or administration of the questionnaire.	Informed consent form for patients in sub-study 1, page 5, Payment
How long is an interview planned to last? Is there a timeframe for the interview?	There is no timeframe for the interviews. For the qualitative phase, it is supposed to last up to 45 minutes. And for the quantitative phase, the administration of the questionnaire will take approximately no more than 60 minutes. This information is added in the protocol. This information can also be seen	Lines 206-207, 274<br><br><br>Informed consent form for patients in sub-study 1, page 2

	in the patient information sheet.	
**Reviewer#3**		
Although the manuscript recognizes the importance of restructuring health systems with greater empowerment of patients, involvement of their families and the communities in the care, the perspectives of family members are not taken into account in this study.	Thanks for this comment. Family members have not been considered for interviews. We recognize that could be of great help to have a broader view.<br><br>Nevertheless, in this particular study, we have chosen the healthcare providers and the patients as it was showed that their perspectives could help build an adequate diabetes care system.<br><br>Reference: Pun SP, Coates V, Benzie IF. Barriers to the self-care of type 2 diabetes from both patients' and providers' perspectives: literature review. J Nurs Healthc Chronic Illn . 2009 Mar ;1(1):4–19.	Lines 114-115
There is a high risk for selection biases, which have also been described in the manuscript.	Thanks for the comment. Selection bias mainly concerns the qualitative component of the study. The purpose of the study is to describe the participants' perspectives on ways to improve glycaemic control. We assume that we will have advantage to find out information-rich patients about the glycaemic control. And that the purposive sampling approach described will ensure to reach them.	No change
**Abstract**		
I would suggest presenting the numbers on T2DM patients with poor glycaemic control in Kinshasa instead of reporting 'a large proportion of patients'.	Thanks for the comment. We have added estimated prevalences of poor glycaemic control in our setting (as described in previous studies).	Line 40

I would suggest providing more details on the qualitative study as part of sub-study 1 (e.g. in-depth review).	We are grateful for the comment. We have added more details of the sub-study 1 in the abstract. The revised description stated that "A minimum of 20 purposively selected patients will participate in the qualitative study that will involve in-depth interviews about their perspectives on glycaemic control". Therefore, the sampling method, the data collection tool and the objective are added.<br><br>The data analysis is also described.	Lines 49-50<br><br>Lines 52-53
**Introduction**		
For non-expert readers in the field: could you elaborate on/provide more details or examples of the adjustments patients with diabetes are required to make, as well as on the definition of glycaemic control (this is not mentioned until the methodology part of the paper (HbA1c < 7%))?	Details on the adjustments have been included in the text.<br><br>Definition of glycaemic control included in the introduction.	Lines 71-73<br>Lines 598-602: Reference 5<br><br>Lines 85-87
50% of patients achieve glycaemic control worldwide. Does this number also include T1DM?	This number only consists of patients with type 2 diabetes as specified in the text.	No change
Could you provide more details on why poor glycaemic control in T2DM patients varies across settings and are complex?	Details have been added between parentheses for each of the characteristics	Lines 77-82
It is unclear whether the reported prevalence of diabetes in the DRC (4.8%) includes both T1DM and T2DM.	We are grateful for this comment. The prevalence of 4.8% reported if for both forms. To be more specific, we have added that the percentage of T2DM in the diabetes population is about 92% as found in one study. The reference has been added.	Line 85
In the following sentence, it is not clear to me what 'the development' refers to: 'The burden of diabetes is a real hindrance to the development of both patients and their families, and also of the health	Thanks for this comment. For better understanding, the sentence has been changed as follows: 'The burden of	Lines 91-93

system oriented towards other priorities with limited resources’	diabetes is a real hindrance to the wellness of both patients and their families, and the effectiveness of the health system oriented towards other priorities with limited resources’	
**Material and methods**		
**Questions related to the qualitative phase**		
The manuscript describes the interview of 20 patients until thematic saturation is reached. Patients are selected purposively with both good and poor glycaemic control. Will a blood sampling be performed before the interview to determine the HbA1c level, or how is the glycaemic control prior to the interview defined? How is this number (20 interviews) distributed across the patients with good and poor glycaemic control?	We are grateful for the comments and questions raised. For the qualitative component of the sub-study 2, patients will be selected independently of their glycaemia control status. A blood sample will be taken from participants for HbA1c assay. But this exam will not be available the same day. We will report on the distribution of poor versus good glycaemic controlled patients in the sample.	Lines 207-209
Is it necessary to interview T2DM patients with good glycaemic control, since the research objective is to define drivers for poor glycaemic control? Or is another objective to define drivers for good glycaemic control as well?	Yes, it is necessary to interview both good and poor controlled patients on drivers of poor glycaemic control. Getting the perspectives from these two groups will help to broader the view on the phenomenon. We assume that the control of glycaemia is not definitive and even controlled patients have been uncontrolled at one point of their disease and could contribute to the understanding of poor glycaemic control.	No change
Is there a difference made between patient with treatment < / > 7 years (cf. quantitative phase)?	Yes, a difference will be made between patients according to treatment duration.<br>We have added a variable for duration treatment in the questionnaire.	See S2 Appendix, page 7, section 23e
Are the interviews performed in 1 of the 66 centers (which one(s))?	No, the interviews will not be conducted in 1 center.	Lines 174-177

	These will be conducted in the centers selected for this study. Their total number is 20.	
How much time will the interview approximately take?	The interview will approximately take up to 45 minutes. The information has been added in the manuscript and in the Informed consent form for patients in sub-study 1	Lines 206-207<br><br>Informed consent form for patients in sub-study 1, page 2.
**Questions related to the quantitative phase**		
The protocol describes an interview procedure, which, in my opinion, is confusing since validated questionnaires are used.	Aligning to reviewer' comment, we will use the term "administration of the questionnaires" instead of "interviews".	Lines 258, 271-274
Since only 20 centers are selected to recruit patients, it seems to me the multicentric study does not comprise 66 centers.	No, the multicentric study will not comprise all the 66 centres. We will use sampling proportional by the size of diabetic patients attending the healthcare facilities to select 20 centres where the study will take place.	No change
Is there a specific reason why the participants will not fill in the questionnaires themselves, but the researchers ask the questions? Will this not create an interpretation bias? Can the questionnaires be completed online by the participants in their home situation?	Considering our setting, very few patients have access to email or the internet to be able to complete the questionnaire through online. This will also introduce a selection bias, if used. The level of education of our patients is also a big hindrance to self-administration of the questionnaire by patients. There is a risk of interpretation bias, which has been identified and will be prevented by the training of data collectors.	Line 387
Why are the characteristics of diabetic disease not collected from the patient's medical file?	The characteristics of diabetic disease are primarily sought from the participants. We use patients 'medical to verify the information given.	Lines 288-289

How much time will the completion of the questionnaires take? It seems a lot of information is asked (18 pages); is this all necessary information related to the research objective?	The time to complete the questionnaire will approximately be of no more than 60 minutes. Once the data collector is familiar with the questionnaire, the time to complete it is shortened.<br><br>All the information sought is related to the research objective.	Line 274
Sub-study 2		
Patients will be selected if they have been followed for at least six months for their diabetes and if they have integrated the model of care offered to patients with diabetes. It is not clear what this 'model of care' comprises.	A model of care broadly defines the way health services are delivered. It comprises the caregivers (doctors, nurses), process of care (diagnostic, treatment, follow-up), access to care, and availability of resources.	No change
**OTHER**	We will use SATA 17 rather that STATA 16 for data analysis	Line 371
	List of references updated with inclusion of new references	References number 5, 26, 30, and 51
	One more element added for enhancing the credibility of the study	Lines 223-224
	More details are added for the selection of patients in the qualitative phase of the sub-study 1	Lines 183-186
	More details have been provided about data analysis in qualitative component of the sub-study 1	Lines 215-218
	More details are provided in the selection of the patients and healthcare providers in the sub-study 2	Lines 412-415
	More details have been provided about data analysis in the sub-study 2	Lines 432-436
	Changes have been made in the Authors contributions section	Lines 566-573
	The quantity of veinous blood to collect is 2	Line 359

	millilitres instead of 5 millilitres.<br>This information appears in the manuscript and the Informed consent form for patients in sub-study 1.	Informed consent form for patients in sub-study 1, page 2
	Language editing for grammatical errors.	

Please do not hesitate to contact me should you wish to obtain more information.

Principal Investigator

Dr FINA LUBAKI Jean-Pierre
